# NBCA-Lipiodol Mixture Embolization of Persistent Urine Leakage After Orthotopic Neobladder Formation: Techniques and Outcomes

**DOI:** 10.3389/fsurg.2022.844588

**Published:** 2022-04-27

**Authors:** Jang Hee Han, Hyeong Dong Yuk, Jin Woo Choi, Ja Hyeon Ku

**Affiliations:** ^1^Department of Urology, Seoul National University Hospital, Seoul, South Korea; ^2^Department of Urology, Seoul National University College of Medicine, Seoul, South Korea; ^3^Department of Radiology, Seoul National University Hospital, Seoul National University College of Medicine, Seoul, South Korea

**Keywords:** glue, embolization, neobladder, technique, bladder cancer, anastomotic leak

## Abstract

**Objective:**

To show the effective and successful technical approach of percutaneous embolization for persistent urine leakage that occurred after orthotopic neobladder formation.

**Materials and Methods:**

We retrospectively reviewed patients who underwent percutaneous embolization of N-butyl cyanoacrylate (NBCA) and lipiodol mixture after orthotopic neobladder formation at the Seoul National University Hospital (Seoul, Korea) from 1 January 2018 to 31 December 2020.

**Results:**

Among total of 182 patients of neobladder formation, five patients (four males and one female) were enrolled in this study, and their median age was 61.0 years (interquartile range (IQR): 42.5–69.5 years). All the patients showed persistent urine leakage at the neobladder-urethral anastomosis site and percutaneous drainage was primarily performed. The median time to perform percutaneous embolization was 40 days (IQR: 31.5–71.5 days) postoperatively. Elective two-staged embolization was performed in three cases for large diameter with a large fluid-filled cavity, while re-embolization was needed for delayed recurrence of urine leakage in two cases. Complete resolution of urine leakage was seen in all the cases and the median time to leakage closure was 55 days (IQR: 27.5–82.5 days). The median follow-up period after leakage closure was 26 months (IQR: 15.5–36.4 months), and embolization material-related bladder stone was a noticeable complication (two cases) during follow-up, which was removed endoscopically within 1 year after embolization. All patients' quality of life (EQ-5D-5L score) was well-maintained during the entire period.

**Conclusions:**

Persistent urine leakage after neobladder formation can be effectively managed with percutaneous embolization of “dumbbell technique” by reinforcing the closure of leakage tract from inner opening to the outer opening even for large diameter (>1 cm).

## Introduction

Convincing evidence suggests that compared to the non-continent conduit method, continent orthotopic neobladder offers a better quality of life (QoL) and greater physical function while having comparable oncologic outcomes (1–4). Despite the promising results, the worldwide trend of orthotopic neobladder performance is currently in the stationary phase, and it is at least partly due to considerable early phase complication rates in orthtotopic neobladder patient group (3, 5, 6).

As Hautmann et al. demonstrated, early complications after orthotopic neobladder are high, approximately reaching 40% (7), and more than 10% of patients required early re-operations. Among them, about 5% of patients are known to experience persistent urine leakage (PUL) which usually occurs at the ileourethral anastomosis site (8). In the recent review of radical cystectomy complications, urethral anastomotic leaks in orthotopic neobladders were reported to reach a rate of 25% in the first 90 days (9). Although most are minor leakages that can be treated by conservative management, often we encounter uncontrollable PUL in certain patients who inevitably experience deterioration of overall QoL and surgical repair becomes typically difficult (10).

As an alternative to surgical treatment, endoscopic or percutaneous embolization with various tissue adhesives (thrombin, fibrin, and collagen glues) has been used as a feasible treatment option in several studies. Offering a convincing success rate and advantage of minimal invasiveness with lesser pain, better economic suitability, and simple technique, the embolization technique is considered to widen its scope to various conditions of post-operative urinary leakages (11–13). The most commonly used material is N-butyl cyanoacrylate (NBCA) (another name: glue), which is often used as a mixture with lipiodol. This agent functions based on the polymerization and changes into a thin elastic impermeable film that is resistant to stretching, and is originally used in the field of vascular embolization, such as arteriovenous malformations, gastric varices, or hemorrhage upon vessel rupture (14). In the field of urology, NBCA is most commonly used for percutaneous obliteration of urinary leakage after partial nephrectomy (15), but attempts for closure of the vesicovaginal fistula, vesicosigmoid fistula, or neobladder-urethral PUL have also been identified in the previous studies (13).

Herein, we demonstrate our initial experience of NBCA (glue) and lipiodol mixture embolization to the PUL occurring at neobladder-urethral anastomosis site. This is the first study using the “dumbbell technique” through a percutaneous approach with a 100% success rate in resolving neobladder-urethral PUL that occurs in the early post-operative period.

## Materials and Methods

### Ethics Approval and Informed Consents

This study was approved by the Institutional Review Board (IRB) of Seoul National University Hospital (IRB no. 2109-075-1254). Informed consent was waived owing to the retrospective nature of this study design. The study was performed in accordance with applicable laws and regulations, good clinical practices, and ethical principles as described in the Declaration of Helsinki.

### Patient Population

We retrospectively reviewed patients who underwent percutaneous embolization of NBCA and lipiodol mixture after radical cystectomy and orthotopic neobladder formation at the Seoul National University Hospital (Seoul, Korea) from 1 January 2018 to 31 December 2020. During the period, five patients were included in this study.

### Intervention Technique

**Figure 1:**
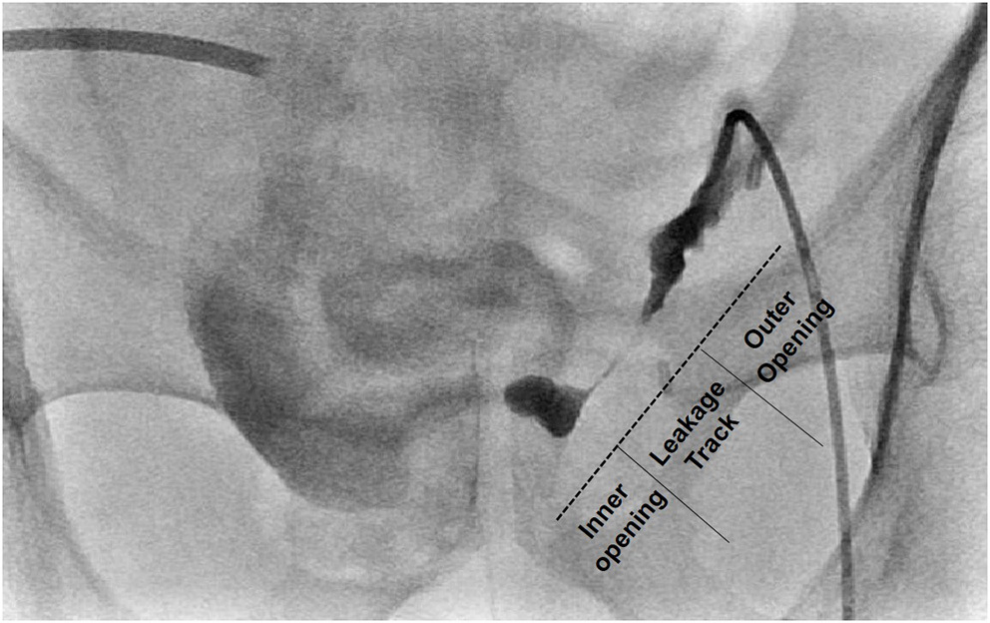
Representative image of “Dumbbell Technique” on fluoroscopic view. NBCA-lipiodol mixture is initially injected onto the leakage tract inner opening, followed by leakage tract, and finally the outer opening of the leakage tract, thereby making it dumbbell-shaped.

Embolization candidate patients underwent percutaneous catheter drainage (PCD) beforehand in the fluid-filled closed cavity that was previously formed by PUL from neobladder-urethral anastomosis site. This preceded process is important because cavity size should be reduced sufficiently to achieve successful embolization. In the embolization method, first, full leakage tract pathway was examined completely using the contrast agent. The inner and outer openings of the leakage tract were well-navigated three-dimensionally and a percutaneous drainage catheter was removed while maintaining the track with the guidewire. Kumpe access catheter was passed through the guidewire and percutaneous embolization was performed using NBCA: lipiodol mixture in the composition of 1:1 or 1:2. Embolization material was initially located on the leakage tract inner opening, followed by along the whole leakage tract, and finally at the outer opening of the leakage tract, thereby making it a dumbbell shape (Figure 1). Then, through the PCD tract, the KMP catheter was re-entered and a contrast test was performed to confirm any leakage. For a relatively small and short leakage track with a small cavity, first attempt success was considered and Kumpe access catheter was removed after the procedure. For a relatively large and long leakage tract with a previously formed large cavity, elective two-staged embolization was planned, and PCD track was maintained with percutaneous drainage catheter or Nelaton tube.

### Collected Parameters

Patient's demographic and clinic-pathological data including gender, age at surgery, disease status (neoadjuvant chemotherapy, pathologic stage), operation type, identification, and management processes of neobladder-urethral leakages, such as cystography imaging, percutaneous drainage catheter insertion, percutaneous nephrostomy catheter insertion, and their maintenance period, embolization related findings, such as material composition, embolization period, follow-up period, and related complications were collected. Additionally, to measure the health-related quality of life score, standardized questionnaire EQ-5D-5L data were collected.

## Results

### Baseline Characteristics of the Study Population

During the study period, a total of 182 patients underwent orthotopic neobladder operation. Among them, five patients (four males and one female) (2.7%) showed PUL and thus included in this study (Supplementary Table 1). The median age of the patients was 61.0 years (interquartile range (IQR): 42.5–69.5 years). The patients' median BMI was 21.8 kg.m^2^ (IQR: 20.0–25.1 kg.m^2^). Four patients underwent neoadjuvant chemotherapy due to high-grade muscle-invasive bladder cancer, while one patient did not. Regarding the operation type, three patients underwent robotic surgery (two intracorporeal pyramid neobladder and one intracorporeal Hautmann neobladder), while two patients underwent open surgery (T pouch neobladder and Studer neobladder), and all patients underwent pelvic lymph node dissection.

### Perioperative PUL Characteristics and Initial Management

All five patients showed PUL at the neobladder-urethral anastomosis site (Table 1). Two patients showed leakage at initial post-operative cystography, while leakage was identified later in three cases. The median time to leakage identification was 18 days (IQR: 11.5–44.0 days). For the conservative management, initially, percutaneous catheter drainage of the fluid-filled cavity was performed followed by percutaneous nephrostomy (PCN) catheter insertion. The median time to decide percutaneous catheter drainage was 20 days (IQR: 13.5–36.5 days) since the operation and it was maintained for a median period of 20 days (IQR: 18.0–66.0 days). Likewise, the median time to decide PCN insertion was 23 days (IQR: 9.5–33.5 days) post-operatively and it was maintained for a median of 49 days (IQR: 28.0–70.5 days). During the treatment, transurethral catheter was maintained for a median of 32 days (IQR: 27.0–35.0).

**Table 1:** Perioperative persistent urine leakage of Neobladder characteristics.

**Patient No**.	**PUL site**	**Initial postoperative cystography finding**	**Time to identification of leakage (days)**	**Time to PCD insertion from operation (days)**	**Time to PCN insertion from operation (days)**	**Transurethral catheter maintenance period (days)**	**PCD maintenance period (days)**	**PCN maintenance period (days)**
1	Neobladder-urethral	No leakage	38	22	7	32	91	84
2	Neobladder-urethral	No leakage	18	15	30	26	19	19
3	Neobladder-urethral	Leakage	11	20	23	35	17	37
4	Neobladder-urethral	Leakage	12	12	12	28	20	57
5	Neobladder-urethral	No leakage	50	51	37	35	41	49
Median (IQR)			18 (11.5–44.0)	20 (13.5–36.5)	23 (9.5–33.5)	32 (27.0–35.0)	20 (18.0–66.0)	49 (28.0–70.5)

*PUL, Persistent urine leakage; PCD,Percutaneous catheter drainage; PCN, Percutaneous nephrostomy; IQR, Interquartile range*.

### Embolization Characteristics and Related Findings

**Figure 2:**
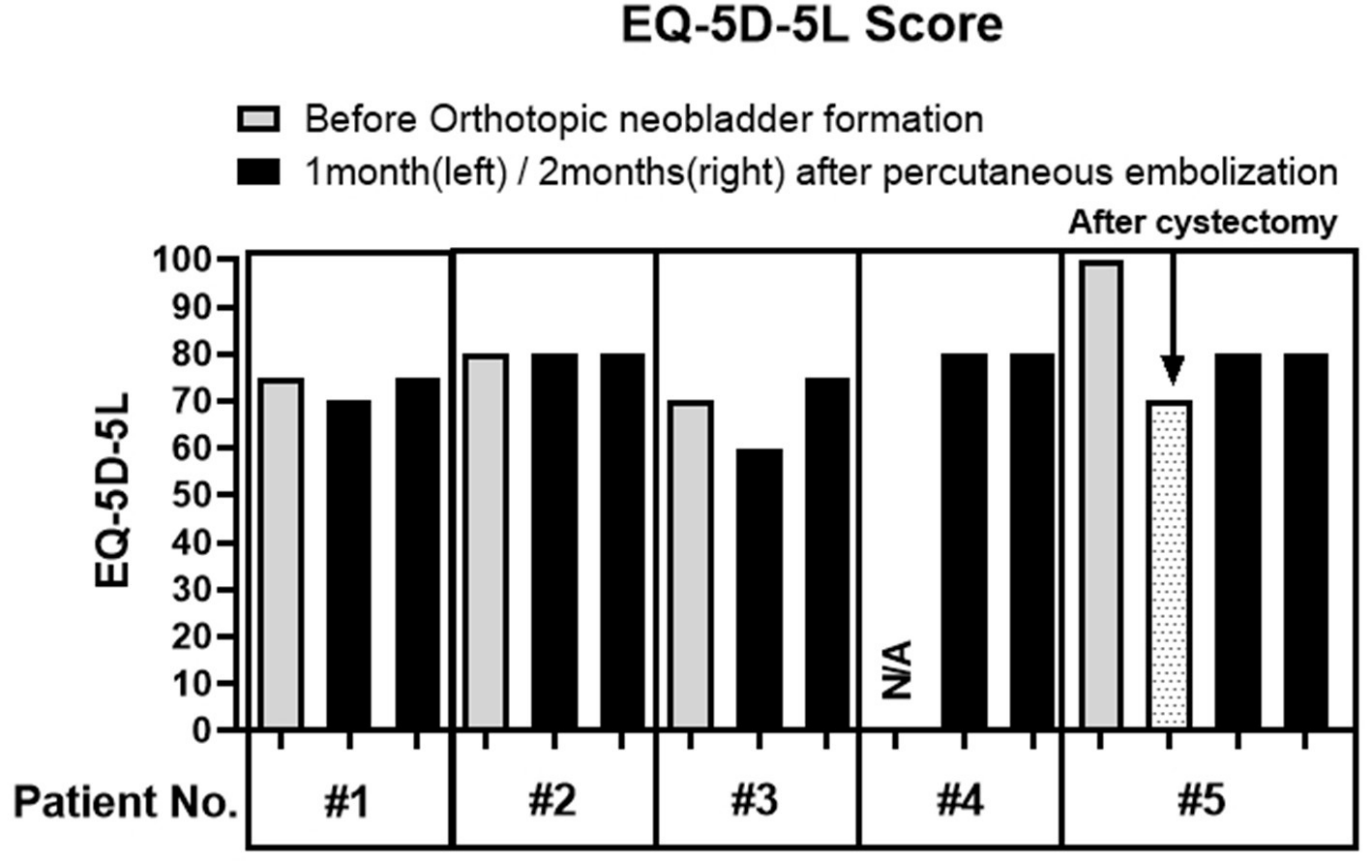
Quality of life score measurement using EQ-5D-5L questionnaire. Patients were asked to fill out standardized quality of life measuring questionnaire, EQ-5D-5L before orthotopic neobladder formation (gray) and at 1 month and 2 months after percutaneous embolization (black). The pre-operative questionnaire was missed in number four patients, while the postoperative questionnaire (dot) was also available for number five patients. None of the patients grossly experienced deterioration of QoL due to percutaneous embolization.

Embolization material composition was NBCA: lipiodol 1:1 or 1:2 mixture. The median time to perform percutaneous embolization since orthotopic neobladder formation was 40 days (IQR: 31.5–71.5 days). Elective two-staged embolization was performed in three cases (60%), while re-embolization was needed for delayed recurrence of urine leakage in two cases (40%). Overall leakage closure was seen in all the cases with the median attempt of two trials (Supplementary Figure 1). The median time to leakage closure since the operation was 55 days (IQR: 27.5–82.5 days) and the median follow-up period after leakage closure was 26 months (IQR: 15.5–36.4 months) (Table 2). Two cases showed NBCA-lipiodol embolization material related to bladder stone, which was removed endoscopically with Holmium laser (Supplementary Figure 2 and Supplementary Table 2). The mean time to bladder stone-like debris formation and its removal was 139.5 and 270 days, respectively All the patients showed a comparable quality of life score on the EQ-5D-5L questionnaire after embolization compared to pre-operative (orthotopic neobladder) results, thereby suggesting procedural advantage (Figure 2).

**Table 2:** Neobladder-urethral leakage tract embolization related findings.

Initial embolization characteristics (*n*)	5 (100)
**Embolization material**	
NBCA: Lipiodol 1:1 mixture (*n*, %)	4 (80)
NBCA: Lipiodol 1:2 mixture (*n*, %)	1 (20)
Median time to embolization from neobladder formation	40 (31.5–71.5)
(IQR) (days)	
2nd-embolization characteristics (*n*)	4 (80)
**Embolization material**	
NBCA: Lipiodol 1:1 mixture (*n*, %)	2 (50)
NBCA: Lipiodol 1:2 mixture (*n*, %)	2 (50)
3rd-embolization characteristics (*n*)	2 (40)
**Embolization material**	
NBCA: Lipiodol 1:1 mixture (*n*, %)	1 (20)
NBCA: Lipiodol 1:2 mixture (*n*, %)	1 (20)
**Re-embolization reason**	
Elective two-staged embolization (*n*, %)	3 (60)
Delayed recurrence of the leakage tract (*n*, %)	2 (40)
Overall leakage tract closure success	5 (100)
Median time to leakage closure (IQR) (days)	55 (27.5–82.5)
Median follow up period after leakage closure (IQR) (months)	26.0 (15.5–36.4)

*NBCA, N-butyl cyanoacrylate; IQR, interquartile range*.

## Discussion

As bladder cancer incidence rises in western countries and accumulating evidences support the survival benefit of early cystectomy (16, 17), the number of radical cystectomy candidates are currently increasing. However, a significantly high complication rate in the early postoperative period makes urologists hesitant to perform orthotopic neobladder. With regard to PUL, the incidence rate was low, accounting for 2.7% in our cases, however, it is usually difficult to treat and a devastating event, thus requiring effective treatment.

In this study, we used the mixture of NBCA and lipiodol as embolization material to treat PUL occurring at the neobladder-urethral anastomosis site. This mixture component is widely verified in vascular embolization with good biocompatibility (14, 18); however, it is not well-utilized in urologic diseases, including urinary fistula or leakage. Compared to NBCA, the mixture of NBCA and lipiodol undergoes faster polymerization and has characteristics of increased viscosity and interfacial tension, which allows the material to reach but not pass through the target (14). Furthermore, the radiopaque characteristics of lipiodol make it easy to approach through percutaneous radiologic intervention (18). Considering the above beneficial effects, we hypothesized that this material would successfully work for closure of the neobladder-urethral leakage tract via a minimally invasive percutaneous approach.

In the previous studies, glue material was mostly injected directly into the fistulous hole either endoscopically or percutaneously (10). This type of approach may be a feasible option for mild cases; however, a more optimized technique is needed to achieve a high success rate for severe cases. We considered that severity is primarily determined by the diameter of the leakage tract opening. Failed cases of the previous study reported having larger than 1 cm of the leakage tract opening diameter (10). In our study, although only two cases (40%) were measurable, they had a leakage tract opening size larger than 1 cm. The intervention time point is another risk factor to be taken into account. Delayed intervention could allow a high chance of complete drainage of the cavity and dryness of the leakage tract, while early intervention may lead to incomplete dryness. The duration of percutaneous catheter drainage maintenance before NBCA embolization was 4.5 times longer (>90 days) in the previous study (10) as compared to our study (median 20 days). Accordingly, the median period of performing embolization was also earlier in our study (median of 40 days since orthotopic neobladder formation). To achieve a high success rate in these high-risk or severe types of patients, we developed our novel technique called the “dumbbell” technique. In this method, we injected embolization material along the whole PUL track by injecting material onto the inner opening, followed by the pathway of the leakage tract, and last the outer opening of the leakage tract, thereby resulting in reinforcement of closure of the urinary leakage tract. As a result, we achieved a 100% success rate in less than 2 months after the PUL was detected (Table 2). In the case of the patients with huge fluid-filled cavity and huge leakage tract opening diameter, elective two-staged embolization was performed with an interval of 1–2 weeks, thereby leading to an average number of twice in achieving a 100% success rate (Supplementary Figure 1). However, the performance of repetitive embolization did not affect QOL deterioration, which is an important point where we can perceive the advantage of minimally invasive percutaneous approach embolization (Figure 2).

However, the present study has certain limitations, primary among which is the small number of patients. With this regard, this study is the feasibility study of unique technique, focusing on technical description and good outcome in neobladder-urethral PUL Further study would be needed for validation of this technique to larger patient cohort. Second, two cases experienced delayed recurrence of PUL with 1 month and 2 months, respectively from the last embolization, thereby indicating that regular follow-up is needed after embolization to thoroughly investigate the recurrence. The positive aspect is that for the delayed recurrence, following reinforcement embolization led to effective complete obstruction of the leakage tract. Another limitation is the frequent occurrence of embolization material-related bladder stones. Two patients underwent endoscopic removal of bladder stones within 1 year after surgery (Supplementary Figure 1). The operative finding was consistent with glue-associated radiopaque debris, co-occurring with bladder stone, which is supposed to have been caused by intravesical embolization material. The optimization of the composition changes of NBCA and lipiodol as well as the amount of material applied to the inner opening should be done since viscosity problems or overdosing could induce the above-mentioned events under the percutaneous approach.

## Conclusions

Neobladder-urethral PUL occurring after orthotopic neobladder formation is often intractable with conservative management, especially when the leakage opening diameter is large with a long tract. Our technique named the “dumbbell technique” employed embolization material of NBCA and lipiodol mixture along the whole urine leakage tract in a dumbbell shape and showed successful complete resolution of PUL.

## Data Availability Statement

The original contributions presented in the study are included in the article/supplementary material, further inquiries can be directed to the corresponding author/s.

## Ethics Statement

The studies involving human participants were reviewed and approved by Institutional Review Board (IRB) of Seoul National University Hospital (IRB no. 2109-075-1254). Written informed consent for participation was not required for this study in accordance with the national legislation and the institutional requirements.

## Author Contributions

JK had full access to all the data in the study and takes responsibility for the integrity of the data and the accuracy of the data analysis and study concept and design. JH, JC, and JK: acquisition, analysis, or interpretation of data, administrative, technical, or material support. JH: drafting of the manuscript. JH and HY: statistical analysis. JC and JK: study supervision. All authors contributed to the article and approved the submitted version.

## Conflict of Interest

The authors declare that the research was conducted in the absence of any commercial or financial relationships that could be construed as a potential conflict of interest.

## Publisher's Note

All claims expressed in this article are solely those of the authors and do not necessarily represent those of their affiliated organizations, or those of the publisher, the editors and the reviewers. Any product that may be evaluated in this article, or claim that may be made by its manufacturer, is not guaranteed or endorsed by the publisher.
