# Supplementary material for: NBCA-Lipiodol Mixture Embolization of Persistent Urine Leakage After Orthotopic Neobladder Formation: Techniques and Outcomes
Source: Front Surg. 2022 Apr 27;9:844588. doi: 10.3389/fsurg.2022.844588 (PMC9091345; doi:10.3389/fsurg.2022.844588)
Supplement: Supplementary file 1 [file Table_1.DOCX]

**­­­­­­­Supplementary Table 1. Patient demographics and pathological variables**

| Patient no. | Sex | Age  (yrs.) | BMI  (kg/cm^2^) | Neoadjuvant chemotherapy | Operation Type | Final Pathologic Stage after radical cystectomy |
| --- | --- | --- | --- | --- | --- | --- |
| 1 | M | 61 | 26.1 | Gemcitabine+Cisplatin | Robot-assisted intracorporeal pyramid neobladder  with Standard template PLND | T3a N0 M0, high grade |
| 2 | M | 55 | 20.7 |  | Robot-assisted intracorporeal pyramid neobladder  with Limited template PLND | T0 N0 M0 |
| 3 | F | 30 | 24.1 | Gemcitabine+Carboplatin | Robot-assisted intracorporeal Hautmann neobladder with Standard template PLND | T3a N2 M0, high grade |
| 4 | M | 63 | 21.8 | Durvalumab+  Gemcitabine+Cisplatin | Open T pouch neobladder  with Standard template PLND | T3a N0 M0, high grade |
| 5 | M | 76 | 19.3 | Atezolizumab | Open Studer neobladder  with Standard template PLND | T0 N0 M0 |

PLND: Pelvic lymph node dissection
