# Supplementary material for: NBCA-Lipiodol Mixture Embolization of Persistent Urine Leakage After Orthotopic Neobladder Formation: Techniques and Outcomes
Source: Front Surg. 2022 Apr 27;9:844588. doi: 10.3389/fsurg.2022.844588 (PMC9091345; doi:10.3389/fsurg.2022.844588)
Supplement: Supplementary file 2 [file Table_2.DOCX]

**Supplementary Table 2. Embolization related complications**

| Complications |  |
| --- | --- |
| No complication (n, %) | 3 (60) |
| Embolization material related bladder stone (n, %) | 2 (40) |
|  |  |
| Treatment of bladder stone |  |
| Cystoscopic bladder stone removal with Holmium laser (n, %) | 2 (40) |
|  |  |
| Mean time to bladder stone formation (days) | 139.5 |
| Mean time to bladder stone removal (days) | 270 |
