# Supplementary figures and images for: NBCA-Lipiodol Mixture Embolization of Persistent Urine Leakage After Orthotopic Neobladder Formation: Techniques and Outcomes
Source: Front Surg. 2022 Apr 27;9:844588. doi: 10.3389/fsurg.2022.844588 (PMC9091345; doi:10.3389/fsurg.2022.844588)

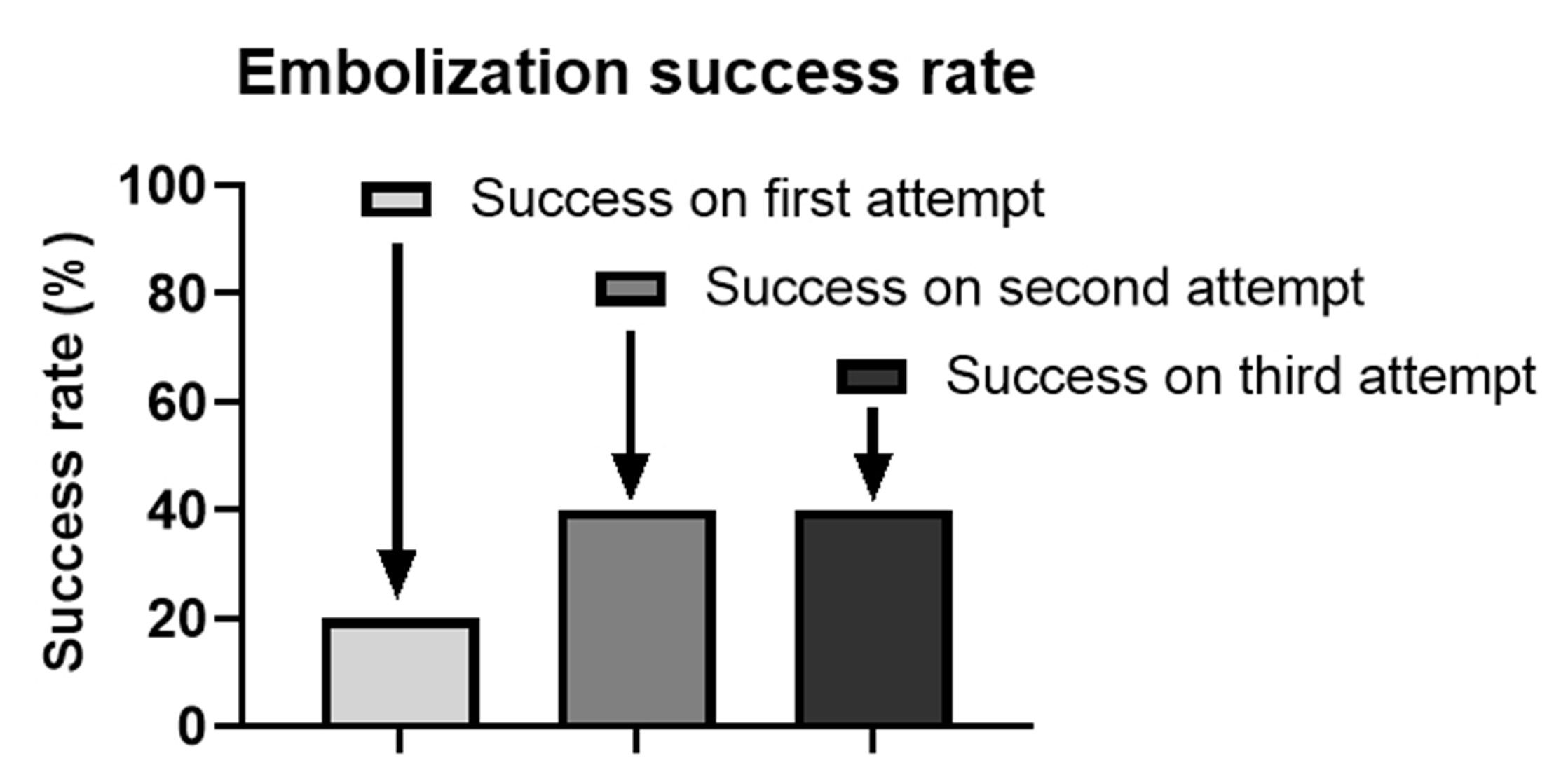

Supplement: Supplementary file 3 [file Image_1.JPG]

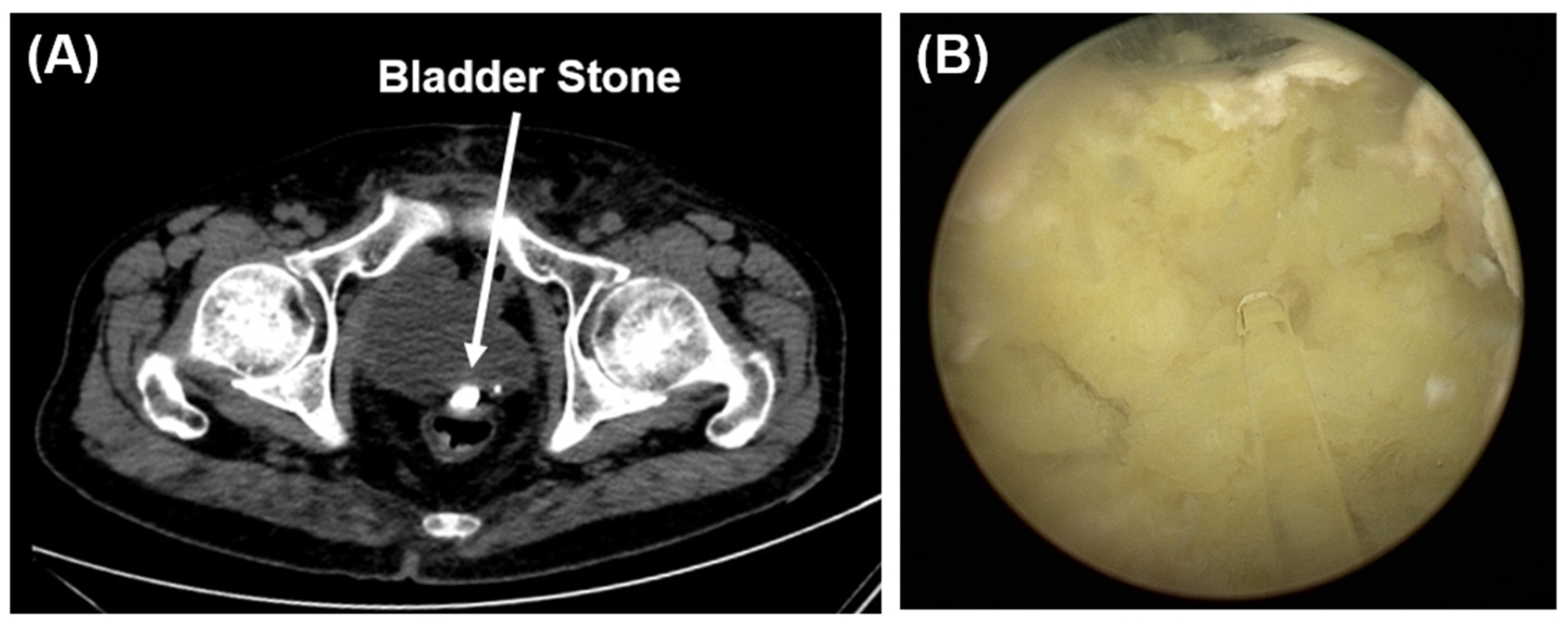

Supplement: Supplementary file 4 [file Image_2.JPG]
